# Opinion Piece: Patient-Specific Implants May Be the Next Big Thing in Spinal Surgery

**DOI:** 10.3390/jpm11060498

**Published:** 2021-06-02

**Authors:** Tajrian Amin, William C.H. Parr, Ralph J. Mobbs

**Affiliations:** 1 NeuroSpine Surgery Research Group (NSURG), Sydney 2000, Australia; tajamin1998@gmail.com (T.A.); parr.will@googlemail.com (W.C.H.P.); 2 Neuro Spine Clinic, Prince of Wales Private Hospital, Randwick 2031, Australia; 3 Faculty of Medicine, University of New South Wales (UNSW), Sydney 2000, Australia; 4 Surgical and Orthopaedic Research Laboratories (SORL), Prince of Wales Clinical School, Faculty of Medicine, University of New South Wales, Randwick 2031, Australia; 5 3DMorphic Pty Ltd., Matraville 2036, Australia

**Keywords:** Three-Dimensional Printing (3DP), custom implant, patient-specific implants (PSI), spinal surgery

## Abstract

The emergence of 3D-Printing technologies and subsequent medical applications have allowed for the development of Patient-specific implants (PSIs). There have been increasing reports of PSI application to spinal surgery over the last 5 years, including throughout the spine and to a range of pathologies, though largely for complex cases. Through a number of potential benefits, including improvements to the implant–bone interface and surgical workflow, PSIs aim to improve patient and surgical outcomes, as well as potentially provide new avenues for combating challenges routinely faced by spinal surgeons. However, obstacles to widespread acceptance and routine application include the lack of quality long-term data, research challenges and the practicalities of production and navigating the regulatory environment. While recognition of the significant potential of Spinal PSIs is evident in the literature, it is clear a number of key questions must be answered to inform future clinical and research practices. The spinal surgical community must selectively and ethically continue to offer PSIs to patients, simultaneously allowing for the necessary larger, comparative studies to be conducted, as well as continuing to provide optimal patient care, thereby ultimately determining the exact role of this technology and potentially improving outcomes.

## 1. Introduction

Three-Dimensional Printing (3DP) refers to the manufacturing method wherein 3D computer aided design (CAD) parts are physically realised through the sequential addition of fine cross-sectional (2D) layers of material. The technology has been widely influential and has seen significant medical application, including producing Patient-specific implants (PSIs) in orthopaedic surgery. PSIs refer to customised implants, tailored to the exact anatomical and surgical needs of each patient, with the key aims of minimising anatomical remodelling and improving implant–bone interface mechanics, osseointegration and surgical outcomes, as well as ultimately improving patient outcomes [1,2,3]. Since the early work of D’Urso et al. [4], 3DP has been extensively applied to spinal surgery, with multiple reports highlighting the utility of spinal biomodels, pedicle screw guides and PSIs [5]. PSIs in spinal surgery have become a particularly promising area, with an increasing number of case reports and small series, particularly of complex cases, emerging over the last 5 years [5,6,7,8,9,10,11,12,13,14]. Though the field is clearly at an early and formative stage, with more data required to validate this technology and its full potential likely yet to be realised, key questions about its future remain. What is the role of 3D-Printed PSIs in spinal surgery? Will every patient get a PSI in the future? Are they only for complex cases? Or will PSIs ultimately be left by the wayside? This article aims to outline the ongoing discussion on PSIs within the spinal surgical community, with particular attention toward current uses and trends.

## 2. Current State of PSI Use in Spinal Surgery

**Figure 1:**
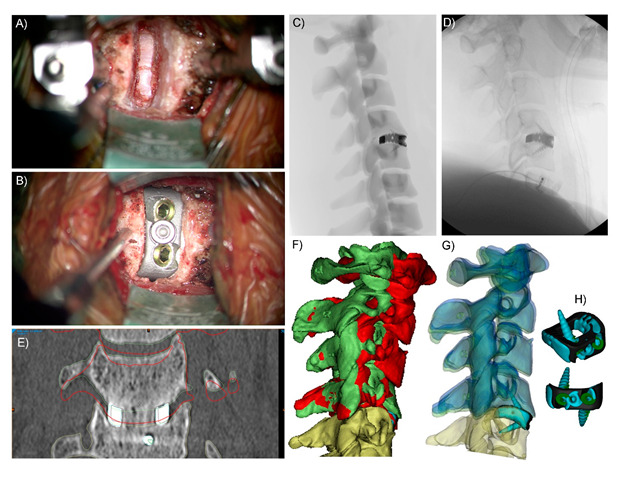
Stand-alone, integral screw fixation Anterior Cervical Discectomy and Fusion (ACDF) Patient-specific Implant (PSI) C4-5. (**A**) Surgical discectomy and preparation of the C4-5 interbody space (**B**) and surgical implantation of the integral screw fixation Titanium alloy PSI. (**C**) Simulated sagittal plane X-ray used intraoperatively to assess implant positioning (e.g., insertion depth), (**D**) actual intraoperative sagittal plane X-ray. (**E**) and three-month postoperative coronal plane CT slice showing fusion bone through the graft window of the Titanium PSI and no discernible subsidence. The red outlines indicate the preoperative position of the C4 vertebra. (**F**) 3D reconstructions of the cervical levels superior to the operative (C4-5) level; red is the preoperative positioning, and green is the achieved (2.5 month) postoperative positioning. (**G**) Translucent 3D reconstructions; green is the achieved (2.5 month) postoperative positioning, and blue is the virtual surgery planned (VSP) positioning. Green positioning is close to the matching blue positioning, particularly when compared to red (preoperative) positioning, which shows good surgical realisation of the plan and that anterior interbody devices can control the postoperative segment angle, as well as (height) distraction. (**H**) Blue (achieved) vs. black (planned) cage positioning within the interbody space; the cage was implanted 0.5–1 mm posterior and to the right of the plan. This was achieved through the use of VSP images (such as **C**) and as a result of the PSI conforming to the patient’s anatomy, thereby auto-locating in surgery into the planned position.

**Figure 2:**
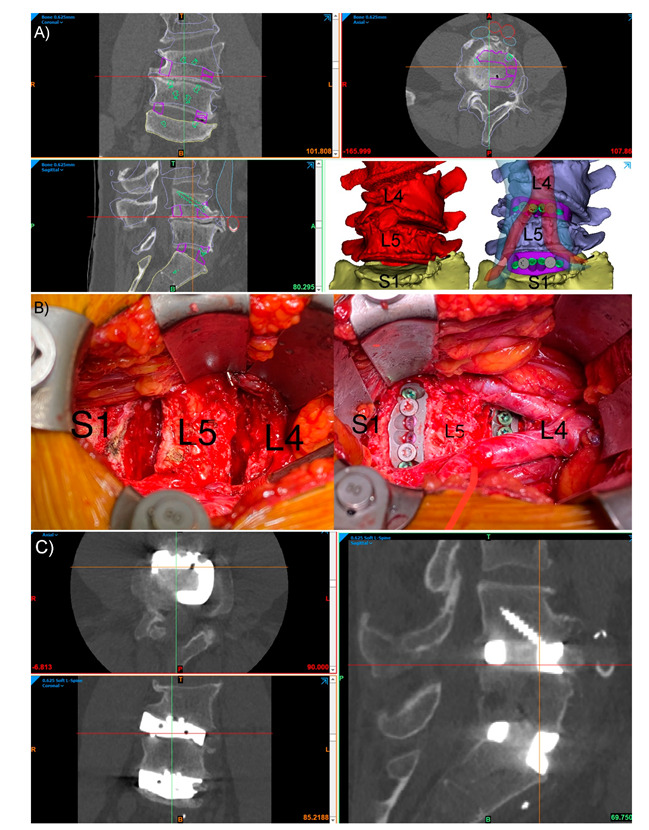
Integral screw fixation, stand-alone Anterior Lumbar Interbody Fusion (ALIF) patient-specific implants (PSIs) L4-5 and L5-S1, in an L4 congenital hemivertebra patient. (**A**) Preoperative CT with planned device (purple outlines), screws (green outlines), vertebral positions (blue outlines) and major vessels (inferior vena cava, blue, and aorta, red). The bottom right panel in (**A**) shows the preoperative pathological anatomy (red) and the planned postoperative state (blue) with translucent aorta (red) and inferior vena cava (blue) shown. (**B**) The intraoperative L4-L5 and L5-S1 discectomies (**left**) and final surgical reconstruction (**right**) with the aortic bifurcation at the L4 level shown. (**C**) Three-month postoperative CT of the construct showing good positioning of the devices, with no evidence of device migration or micromotion, and interbody fusion bone forming through the graft windows of both PSIs.

**Figure 3:**
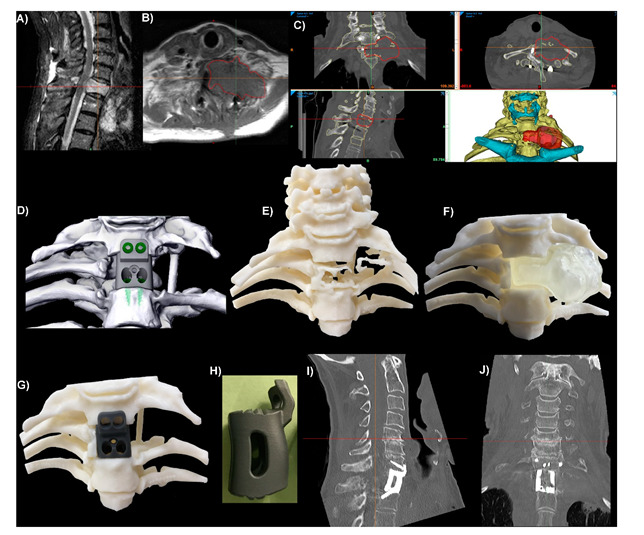
Integral screw fixation thoracic (T1) corpectomy/vertebrectomy patient-specific implant (PSI). (**A**) Sagittal plane MRI slice showing a tumour in the T1 vertebral body. (**B**) Axial plane MRI slice with red outlines showing the tumour. (**C**) CT slices and 3D reconstruction of the anatomy. The hyoid and sternum are shown in cyan. The position of T1 relative to the sternum meant that access to insert screws up into C7 would be difficult, so a custom anterior plate was integrated into the interbody device with anterior–posterior screw trajectories planned for C7. (**D**) Virtual Surgical Plan tumour resection and surgical reconstruction using the PSI and integral screws. (**E**) 3D-Printed ‘biomodel’ of the vertebral and rib bone showing the lytic effects of the tumour on the T1 vertebral and rib bone. (**F**) 3D-Printed biomodels of the vertebral bone with a removable tumour (opaque, colourless). (**G**) Same bone biomodel as (**F**) with the tumour removed and a 3D-Printed resin ‘demo’ PSI in position. (**H**) Sagittal plane viewpoint of the Titanium alloy (Ti_6_Al_4_V) PSI. One-day postoperative sagittal (**I**) and coronal (**J**) plane CT slices of the level showing good positioning and contact between the PSI and bone.

Since the early case reports of Xu et al. [15], Phan et al. [16] and Wei et al. [17] in 2016, there has been a rapid increase in reports of Spinal PSI use. An encouraging development has been the emergence of small case series over the last 3 years, namely the reports from Girolami et al. [6] and Wei et al. [7], indicating a growing acceptance by clinicians, the potential scalability of these technologies, and the transition to higher levels of research. PSIs have now been used to manage a range of pathologies, including infection [11], degeneration [18], malignancy [19] and deformity [9], throughout the spine at the cervical [7], thoracic [19], lumbar [6] and sacral [17] levels. These cases have generally involved highly complex patho-anatomies, with a PSI indicated following the assessment of the surgical team that no commercially available generic or Off-The-Shelf (OTS) implant would provide an acceptable surgical outcome. In these scenarios, OTS implants are often deemed unlikely to produce good outcomes due to a significant implant–bone shape mismatch. Generally, this mismatch will be compensated for through extensive remodelling of the bony anatomy to fit the implant, likely resulting in increased operative time and trauma due to high-speed bone burring, a weakened bony anatomy and possibly increased subsidence risk, as well as a likely suboptimal implant–bone contact area and suboptimal force distribution. Regarding the literature to date, implants have largely been high cervical spine, vertebrectomy, interbody and sacral devices, generally manufactured from Titanium alloy. Figure 1, Figure 2 and Figure 3 illustrate a range of PSI types used in our own practice.

A large suite of custom features have been described, including endplate matching, integral fixation, planned screw trajectories, windows for bone growth and radiographic assessment, variable surface porosity, biomimetic structures and integration with posterior hardware [5,6,11,20]. While authors have frequently emphasised the need for further research, with larger samples and comparative methodologies, as well as the limitations associated with 3DP and PSIs, these early reports have been largely favourabe, and a general appreciation of the promise of this technology is apparent [1,5]. However, the current evidence, at best, may be used to support PSI use only by highly experienced surgical teams in highly selected cases, namely patients with complex patho-anatomies, where the use of an OTS implant is unlikely to produce acceptable outcomes, and who provide informed consent after a comprehensive education, including the overall early stage, unproven and, sometimes, experimental nature of this emerging technology.

## 3. Why Should Spinal Surgeons Use PSIs?

A range of theoretical advantages have been ascribed to PSIs, largely to do with improvements to the implant–bone interface and the overall surgical workflow. The key advantages at the implant–bone interface centre around endplate matching, which refers to the matching of the contacting surfaces of the implant and the patient anatomy. This minimises the need for endplate preparation, thereby preserving bone integrity, improving force distribution and osseointegration, as well as improving the primary stabilisation and minimising stress shielding [1,5,8,21]. The ability to manipulate the surface porosity of Titanium PSIs may additionally enhance osseointegration [21]. An improved surgical workflow is largely thought to be a result of the faster implant fit and reduced need for endplate preparation, as well as the associated preoperative planning and biomodelling (Figure 2 and Figure 3), thereby possibly reducing operative times, blood loss, fluoroscopy use and, ultimately, costs [1,5,20,21]. Custom features may provide specific further advantages. For example, pre-planned screw trajectories and screw lengths may improve the primary stabilisation and reduce the risk of a screw exiting the bone and damaging neurovascular structures [8], as well as possibly reduce the time required to achieve screw fixation. While the current literature is undoubtedly lacking, the growing number of early reports, from multiple authors from multiple centres [5,6,7,8,9,10,11,12], detailing the successful application of spinal PSIs to a range of clinical scenarios is encouraging. 

The characteristic customisability of PSIs may lend them particularly suited to spinal surgery, given the inherently complex anatomy of the spine, consisting of 33 vertebrae with up to seven joints at each vertebral level [22,23,24], as well as the often complex distortion produced by common pathologies, including degeneration, malignancy and deformity [11]. While, in this setting, PSIs are intuitively more likely to be superior tools in comparison to OTS generic options, the customisability of PSIs also provides spinal surgeons with new avenues of combating some of the key challenges they routinely face. Cage subsidence can significantly reverse the surgically gained improvements to disc and neuroforaminal heights, thereby potentially leading to poorer clinical outcomes [25]. Guided by an understanding of the risk factors and mechanisms associated with cage subsidence [25], including cage design and bone quality, this not uncommon postoperative complications may be minimised through careful cage design and the minimal endplate preparation afforded by PSIs [6,7,8]. In cervical surgery, 3DP can allow for patient-specific, truly zero profile implants that may minimise the dysphagia and dysphonia associated with conventional instrumentation [20,26]. 

Spinal reconstructive surgery, particularly as indicated by primary osseous malignancy, often involves significant bone loss and may involve physiologically complex anatomy, such as the high cervical spine. These challenging operating conditions may commonly result in profound instability and instrument-related complications. PSIs have been described as a potential way of combating this by allowing for strong primary stabilisation, filling of the defect and deformity correction [6,7,27]. Figure 3 provides an example of our relevant clinical experience. Wei et al. also suggested that PSIs may be less prone to instrument failure secondary to postoperative radiotherapy, in comparison to the reported issues with conventional reconstructive methods [7]. The ability to tailor implants to the exact size required is also hugely useful in the case of severe, progressed and/or recurrent presentations, where no appropriately large OTS implant may exist [10,11]. PSIs are particularly useful in managing infections, as bone infections can cause large defects, significant bone loss and irregular bone surfaces [11]. While a PSI can fill this defect, as described by Chung et al. [11], and also likely achieve good primary stabilisation, PSIs with antimicrobial properties have also been described. As the surface porosity of these implants would allow for a drug delivery system to be included, the risk of new-onset Surgical Site Infection or recurrent infection can be minimised in these cases, thereby preventing subsequent dismal outcomes [28,29].

## 4. What Are the Issues?

The key issue facing Spinal PSIs is the lack of quality, long-term data demonstrating their utility, safety and superiority over OTS alternatives, likely in terms of patient, surgical and economic outcomes. While PSIs have a longer and more extensive history of application elsewhere in orthopaedics, such as to Total Knee Arthroplasty (TKA), concerningly the current body of evidence, though including some encouraging recent results highlighting the improved precision afforded by PSIs in TKA [30,31,32], does not clearly demonstrate the superiority of PSIs and, thus, fails to validate the theoretical benefits of their use [33,34]. Evidently, significant further research is required in this area, particularly of the longer-term outcomes, as well as in light of emerging evidence regarding the specific preoperative factors affecting surgical outcomes and the importance of careful patient selection [35].

While further research is clearly needed, a number of issues complicate this pursuit. The literature is currently focussed on complex cases, with inherently limited external validity. This limits the extent to which clinicians can apply these early results to clinical scenarios they encounter, as well of researchers to justify larger, comparative studies in less complex, more routine patients. The wide spectrum of patho-anatomical complexity also complicates certain essential analyses, including between PSIs in standard and complex cases, as well as between PSIs and OTS implants in cases of similar complexity. It is clear that a method of describing patho-anatomical complexity, perhaps through a qualitative grade or a quantitative index, is needed to further the literature. Researchers should also ensure that key PSI design and manufacture parameters are clearly reported and that only fundamentally similar PSIs are compared. As PSIs may sometimes make certain operations possible [9], comparisons against an OTS implant which would not have actually been used, are invalid. As other 3DP-related tools are often used alongside PSIs, including biomodels and custom instruments [20], certain analyses will likely be confounded, including assessing the impact of PSIs alone on surgical outcomes. However, given their routinely combined use, simply assessing for the overall impact of a patient-specific procedure may still be meaningful.

The other broad category of issues facing spinal PSIs revolve around the practicality of their use. PSI design and manufacture can be resource-intensive, requiring specialised skills and equipment [1,5]. However, this will likely be less important in the future considering further growth of the literature, growing familiarity and the rapid pace of development in the manufacturing fields, as well as the possible development of user-friendly holistic software solutions [3,8] and the emergence of private companies offering an integrated suite of these services [9]. The inflexibility of PSIs has also been criticised. A number of requirements must be met, including good quality imaging, careful computer processing and relatively short imaging-to-operation times, to ensure that the implants are still patient-specific [21]. Additionally, cancelled or delayed operations, as well as intraoperative positioning and both intended and inadvertent surgical remodelling, may additionally compromise the specificity and insertion of these implants [1,11]. Therefore, a number of PSIs in different sizes are often required to allow for the best fit to be determined intraoperatively [8], and OTS alternatives may also be kept on-hand.

The regulatory environment has been highlighted as a key potential obstacle for surgeons. While likely to evolve, it may be restrictive and challenging to navigate [1,5,9], though this may also be attenuated with time, mounting evidence and wider familiarity. As highlighted by Willemsen et al., the existing regulatory environment may frustrate the current use of PSIs for cases with a degree of urgency, including when dealing with malignancy or spinal instability [9]. This is particularly problematic given that these cases, often with complex and large defects, may stand to greatly benefit from this technology. While Willemsen et al. framed their devices as custom-made or personalised and so avoided the more complex and time-consuming reporting otherwise required for medical devices, these kinds of exemptions, though appropriate for select cases, would represent an abuse of the regulatory process given a sufficient patient volume and so are unsustainable in the long term. As discussed by Mobbs et al., clearly researchers, clinicians and regulators must strike a balance between lax oversight, culminating in unsafe devices being used and excessive restrictions stifling innovation, delaying state-of-the-art care options and denying patients the best management in their clinical context [21]. Table 1 summarises the key considerations for spinal surgeons regarding PSI use.

## 5. Discussion

The growing interest in personalised medicine is clear. Driven by advances in the basic sciences and technology, clinicians are trying to optimise management, eliminate trial and error and, ultimately, improve outcomes. This is particularly evident in medical specialties, as evidenced by the emergence of pharmacogenomics and pharmacodiagnostics in lieu of traditional algorithmic and iterative approaches [36]. Wearable devices for continuous and objective patient monitoring present another excellent example of this technology-driven, highly patient-centred approach [37]. The personalised care paradigm has now increasingly begun to shape the surgical fields [3,38,39,40], with 3DP proving to be a versatile tool.

While some patient-specific 3D-Printed developments, namely biomodels and custom instruments, are likely to persist given the practically negligible potential for serious harm and the reasonable benefits provided in planning, training and education, as well as possibly reducing operative times [20], PSIs present a much greater challenge given their inherently invasive and essentially permanent nature. In short, the stakes are much greater. Ultimately, the turning point for PSIs will likely be the verdict of quality, long-term data investigating their outcomes in comparison to OTS generics. This evidence, alongside economic considerations, particularly with future streamlining of the design and manufacturing process, will likely guide which populations receive PSIs in the future. PSIs may prove to greatly improve outcomes in comparison to OTS alternatives and be sufficiently cost-effective enough to be used for all patients. However, if only a minor improvement to outcomes, or at least noninferiority, is demonstrated, economic considerations will likely guide their use. Routine use is more likely if PSIs do significantly reduce operative times and so result in significant cost savings [20]. Otherwise, they may continue to be used only in select patients to aid with complex cases. 

Regardless, it is clear that a balance must be struck in the interim. Early on, Harrington’s eponymous rods were also patient-specific and used in select cases prior to the transition to larger patient groups, widespread use and acceptance [41]. On the other hand, spinal surgeons and pioneers must not allow a sound theoretical basis, successful application in other fields and encouraging early results to drive unsubstantiated, widespread application, as some argue has occurred with certain Minimally Invasive Spinal Surgeries (MISS) [42]. Further, the possibility of unforeseen implant-related complications must not be discounted, either due to issues in the planning and implementation of a PSI [6] or inherent to the implant design or material [11,18,43].

Future areas of interest include optimising materials and custom features [5,10,44], ideally with the aims of further improving outcomes and continuing to pursue solutions to problems facing the spinal surgical community. Can patient-specific arthroplasty implants for Total Disc Replacements be produced? Can PSIs be made to suit MISS, allowing for smaller incisions and less retraction? Can devices be optimised for particular surgical techniques, including for the degrees of access they allow and the accessory instrumentation they may include [45]? Can PSIs, in combination with virtual surgery planning, reduce the risks of spine surgery sufficiently to enable better surgical outcomes by less experienced surgeons/surgical teams? Can tissues be bioprinted to combat specific operative challenges and further improve outcomes [46]? For example, can bioprinted disc substitute material or an annular defect repair patch combat post-microdiscectomy height loss or recurrent disc herniation, respectively? Research in these areas will help to distinguish the exaggerated and overly optimistic predictions of the benefits and potential uses of 3DP, PSIs and associated technologies from the realistic, practical and clinically relevant applications that researchers and clinicians should explore. In summation, the great potential of this technology is clear, but further work is required to substantiate this. The spinal surgical community must ethically apply this technology to more patients and for more indications, ultimately allowing for the larger, comparative studies and scientifically sound comparisons to be made which will shed light on the role of this technology, shape the regulatory environment and, ultimately, potentially improve outcomes. 

## Figures and Tables

**Table 1:** Summary of the key advantages and disadvantages, including largely theoretical points, associated with patient-specific implant use by spinal surgeons.

Advantages	Disadvantages
Easier Implantation	Lack of Quality Data
Minimal Endplate Preparation	Research Challenges
Improved Device–Bone Load Distribution	Skilled Labour and Equipment Requirements
Improved Primary Stabilisation	Increased Preoperative Planning
Range of Customisable Features	Reduced Intraoperative Flexibility
Enhanced Osseointegration	Multiple Implants Need to be Produced Per Case
Minimised Operative Time	Off the Shelf Devices Often Also Kept on Hand
Tailor to Specific Operative Challenges and Clinical Scenarios	Challenging Regulatory Environment
